# Anti-Idiotypic Antibody as a Booster Vaccine Against Respiratory Syncytial Virus

**DOI:** 10.3390/vaccines13010035

**Published:** 2025-01-02

**Authors:** Shreya Mukhopadhyay, Ioannis Manolaridis, Christopher Warren, Aimin Tang, Gregory O’Donnell, Bin Luo, Ryan P. Staupe, Kalpit A. Vora, Zhifeng Chen

**Affiliations:** 1 Infectious Diseases and Vaccine Research, Merck & Co., Inc., Rahway, NJ 07065, USA; shreya.mukhopadhyay@merck.com (S.M.); christopher.warren@merck.com (C.W.); aimin_tang@merck.com (A.T.);; 2 Protein and Structural Chemistry, Merck & Co., Inc., Rahway, NJ 07065, USA; ioannis.manolaridis@merck.com; 3 Quantitative Biosciences, Merck & Co., Inc., Rahway, NJ 07065, USA; gregory_odonnell@merck.com (G.O.); bin_luo2@merck.com (B.L.)

**Keywords:** Respiratory Syncytial Virus (RSV), RSV Fusion protein (RSV F), anti-idiotypic antibodies (anti-ID), structural mimicry, cryo-EM, epitope-specific immunization

## Abstract

**Background/Objectives:** The respiratory syncytial virus (RSV) is a major cause of lower respiratory tract infections in children and adults. With nearly everyone infected by the age of five, there is an opportunity to develop booster vaccines that enhance B-cell immunity, promoting potent and broadly neutralizing antibodies. One potential approach involves using anti-idiotypic antibodies (anti-IDs) to mimic specific antigenic sites and enhance preexisting immunity in an epitope-specific manner. RB1, a monoclonal antibody (mAb) that binds to site IV of the RSV fusion (RSV F) protein, is a potent and broadly neutralizing against RSV A and B viruses. It is the precursor for MK1654 (clesrovimab), which successfully completed a Phase III clinical trial. **Methods:** In this study, we isolated two anti-IDs, 1A6 and 1D4, targeting RB1 CDR regions, demonstrating that 1A6 competes fully with RSV F in binding to RB1. **Results:** We resolved the RB1-1A6 and RB1-1D4 Fab-Fab complex structures and proved that 1A6 mimics the RSV F site IV better than 1D4. In an immunogenicity study, mice primed with RSV F and boosted with 1A6 Fab showed a site IV-specific antibody response with a concurrent increase in RSV virus neutralization. **Conclusions:** These results suggest that anti-IDs could be potentially used as booster vaccines for specific epitopes.

## 1. Introduction

The idiotypic (ID) antibody network theory, proposed by Neils Jerne [1,2,3], suggests that the immune response to a specific antigen can be regulated by idiotypic antibodies (Ab1) and anti-idiotypic antibodies (anti-ID or Ab2). Idiotope refers to a single antigenic determinant on the variable regions of antibody molecules [4,5,6]. When animals or patients receive an Ab1, a specific group of anti-IDs (Ab2) is produced, recognizing an Ab1 within the antigen binding site and carrying an internal image of the original antigenic epitope. The antigen-binding site is located on the tip of the antibody Fab portion. Ab2 can further generate immune responses to produce anti-anti-IDs (or Ab3) [4], which in turn can function similarly to Ab1. This Ab2 can potentially be used as a site-specific booster vaccine for antigens with multiple antigenic sites/epitopes. While there are no US FDA-approved anti-ID vaccines, some studies have explored their use for infectious diseases in animals [7,8,9]. Additionally, there are therapeutic biologics approved by the US FDA, such as Dinutuximab [10] for high-risk neuroblastoma, and Racotumomab (Vaxira) [11] approved for Non-Small Cell Lung Cancer patients in recurrent or advanced stages (IIIB/IV) in Cuba and Argentina.

RSV (Respiratory Syncytial Virus) is a leading cause of lower respiratory tract disease in infants and seniors. The FDA has approved several products as anti-RSV prophylactics based on the age group, including two monoclonal antibodies (mAb), Palivizumab [12] and Nirsevimab [13], and three vaccines, Arexvy [14,15], Abrysvo [15], and mRESVIA [16]. These products target the RSV F (Fusion) protein, a surface glycoprotein that plays a crucial role in viral entry and fusion with host cells, leading to infection. The mature RSV F glycoprotein exists in a metastable pre-fusion conformation (preF), which then converts into a more stable postfusion conformation (postF) after interaction with the host cell membrane. RSV preF is the principal target for neutralizing activity in human serum and has been a desirable target for vaccines and antibody-based therapies.

The RSV F protein is known to have at least six immunogenic sites that can induce immune responses [12,17,18,19,20,21], which include the antigenic sites Ø (Zero), I, II, III, IV, and V. Antigenic sites Ø [17] and V [22] exist exclusively in the preF conformation of the RSV F protein. On average, the antibodies against these sites have also shown higher neutralization potencies [22]. Previous studies showed that Site IV is one of the most conserved sites within the RSV F protein of both RSV A and RSV B viruses [23,24,25]. We have isolated site-IV-specific human mAb RB1 [23], a potent, broadly neutralizing mAb for RSV A and B viruses, which served as the parental molecule for MK1654 (clesrovimab). We hypothesized that an anti-RB1 ID antibody might mimic its epitope on antigenic site IV of RSV F and could be used as a site-IV-specific booster vaccine. In a recent study, we discovered two anti-ID mAbs, 1A6 and 1D4, targeting RB1 using phage-display panning [26]. These two anti-ID mAbs compete with the binding of RB1 to RSV preF differently [26]. We determined high-resolution cryo-electron microscopy (cryo-EM) structures of RB1 Fab binding to 1A6 and 1D4 Fabs, elucidating the differences in the binding mode of these antibodies to RB1. We further demonstrated that 1A6 better mimics the RSV preF site IV binding to RB1 than 1D4. From the methods perspective, these human Fab-human Fab structures represent a cryo-EM achievement due to their small complex size (~100 kDa) and inherent particle pseudosymmetry. There are a few more structures of Fab-Fab complexes solved by X-ray crystallography [27,28,29], and one recent report about mouse Fab-human Fab structure solved by Cryo-EM [30]. We tested 1A6 Fab as a booster vaccine in mice primed with RSV preF and found that 1A6 increased RSV neutralization titers and site-IV-specific RSV F antibody response. These findings indicate that anti-IDs might have the potential to serve as epitope-specific booster vaccines for infectious diseases.

## 2. Materials and Methods

### 2.1. Preparation of RSV Fprefusion F Protein and the Anti-IDs

The method for preparing RSV prefusion F protein was described previously [17,31]. Briefly, a plasmid encoding RSV prefusion F protein (DS-Cav1) based on RSV A2 sequences was used to transfect Expi 293 F cells (Thermo Fisher Scientific, Waltham, MA, USA). F protein was purified from cell culture supernatants using Ni-Sepharose chromatography (GE Healthcare, Chicago, IL, USA) and further purified using gel filtration chromatography (Superdex200, GE Healthcare, IL, USA). The heavy and light chain sequences of RB1 IgG or 1A6, 1D4 Fabs, and IgGs were sub-cloned into a pTT5 vector for CHO-3E7 cell expression. The CHO-3E7 cells were grown in serum-free FreeStyle CHO Expression Medium (Thermo Fisher Scientific, Waltham, MA, USA). The recombinant plasmids encoding heavy and light chains of each antibody were transiently co-transfected into CHO-3E7 suspension cell cultures. The supernatants collected after 6 days were applied to a MabSelect SuReTM LX column (Cytiva, Marlborough, MA, USA) and CaptureSelect™ CH1-XL Affinity Matrix (Thermo Fisher Scientific, Waltham, MA, USA) for purification. The purified antibodies were buffer-exchanged to 1 × PBS, and QC tested by SDS-PAGE and Western blot analysis (GenScript, Piscataway, NJ, USA) (Supplementary Figure S1).

### 2.2. RB1 Scrambled ELISA with Anti-IDs

RB1 HCDR3, LCDR3, or both sequences were shuffled such that the scrambled variant maintains the same building blocks as the WT protein but rearranges the amino acid order in which they appear. This allowed us to assess the importance of RB1 HCDR3, LCDR3 (GenScript, Piscataway, NJ, USA), or both in the interaction with 1A6 Fab or 1D4 Fab (GenScript, Piscataway, NJ, USA), compared to that with the RB1 WT (GenScript, Piscataway, NJ, USA). 1A6 or 1D4 anti-ID Fab was coated on the ELISA plate at 1 µg/mL overnight, followed by blocking using Blocker™ Casein buffer (Thermo Fisher Scientific, Waltham, MA, USA) for 2 h at room temperature. RB1 WT or the three mutants mentioned above were allowed to bind to the 1A6- and 1D4-coated plates. RB1 WT and three other CDR3 scrambled constructs were added as a primary antibody and titrated from 1000 ng/mL to 0.05 ng/mL as an 11-point dilution with 3-fold dilution. The plates were washed five times with PBS-T (200 µL/well) using Titertek M96 Washer between all steps. Anti-human IgG Fc-HRP Antibody at 1:20,000 was used to interact with the Fc region of the primary antibody for 30 min. The plates were developed using the TMB substrate solution (Thermo Fisher Scientific, Waltham, MA, USA) for four minutes, and the enzymatic reaction was stopped by the TMB STOP solution (Thermo Fisher Scientific, Waltham, MA, USA). Two technical replicates of the experiment were performed, and the mean and standard deviation were calculated.

### 2.3. Biacore Binding Affinity Studies of RB1 with Anti-IDs

The binding of 1A6 or 1D4 Fab to MK-1654 IgG was analyzed on a Biacore 8K+ instrument using a Series S Protein A sensor chip (Cytiva, Marlborough, MA, USA) to capture MK-1654 IgG at 1.34 nM for 120s at 10 µL/min, achieving a capture level of approximately 110 RU. Subsequently, 1A6 or 1D4 Fab were added to both flow cells at increasing concentrations (0 nM, 0.4 nM, 1 nM, 4 nM, 8 nM, 16 nM, 32 nM, 64 nM, 128 nM), with a maximum surface density of 40–70 RU, and a contact and dissociation time of 60 s each at a flow rate of 30 µL/min. Nine consecutive cycles of association and dissociation were allowed to occur, with two serial identical regeneration cycles in between using glycine at pH 1.5. Data analysis utilized BIAevaluation software (GE Healthcare, version 4.1), deriving association (*k*_on_) and dissociation (*k*_off_) rate constants via global fitting analysis, assuming a 1:1 stoichiometry. The dissociation constant (K_D_) was determined from the ratio of the rate constants (*k*_off_ /*k*_on_) as previously described [32].

### 2.4. Cryo-EM Sample Preparation, Data Acquisition, and Processing

#### 2.4.1. Grid Preparation and Data Collection

The 1A6-RB1 and 1D4-RB1 Fab-Fab complexes were prepared by applying a 3 μL sample of the purified complex onto glow-discharged EM grids, blotting for 3.5 s, and rapidly plunge-freezing in liquid ethane using a Vitrobot Mark IV (Thermo Fisher Scientific, Waltham, MA, USA) at 95% humidity and 4 °C. For 1A6-RB1, a glow-discharged R2/1 300 mesh holey NiTi grid (Single Particle LLC, San Diego, CA, USA) was utilized at a concentration of 3 mg ml^−1^, while for 1D4-RB1, a glow-discharged R2/1 300 mesh holey carbon copper grid (Quantifoil Micro Tools GmbH, Jena, Germany) was employed at a concentration of 1 mg ml^−1^. The vitrified samples were stored in liquid nitrogen before imaging on a 300 keV Titan Krios cryoelectron microscope equipped with an energy filter (Gatan Inc., Pleasanton, CA, USA) and a post-GIF Gatan K3 summit direct detector. Image acquisition was performed at a calibrated magnification of 105,000× corresponding to 0.815 Å per physical pixel (Supplementary Figure S2). The dose fractionation was set to 1.06 e^−^/Å^2^/frame with a total of 40 frames, resulting in a total dose of 42.5 e^−^/Å^2^. Data collection was fully automated using EPU in the Gatan Imaging Suite (Gatan Inc., Pleasanton, CA, USA), with a nominal defocus range from −0.8 to −2.2 μm, and Image Shift was utilized for improved throughput. In total, 11,590 and 10,325 videos were collected for 1A6-RB1 and 1D4-RB1, respectively (Supplementary Figures S3 and S4).

#### 2.4.2. Cryo-EM Data Processing

The cryo-EM map processing workflow using CryoSPARC [33] (version 4.4.1) included map sharpening, and 2D and 3D classification. In total, 11,590 and 10,325 videos of 1A6-RB1 and 1D4-RB1 complexes were curated (Supplementary Figure S3 and S4). Motion correction using MotionCor2 [34] addressed motion artifacts, and CTFFIND4 [35] improved map resolution by detecting defocus parameters and addressing blurring. Template picking and iterative reference-free 2D classification in CryoSPARC [36] generated initial class averages showcasing structural features. An Ab-initio 3D classification [33,36] identified distinct conformations within the dataset. Multiple rounds of 3D classification using the “decoy” method segregated non-relevant decoy particles, enabling accurate 3D classification. The resulting electron density map underwent map sharpening using the EMReady density modification program [37] for improved visualization. These cryo-EM maps were utilized for model building, refinement, and validation, enhancing understanding of the macromolecular structures within the two Fab-Fab complexes.

#### 2.4.3. Model Building and Refinement

Chains M + N of the RB1-RSVF crystal structure (PDB 6OUS) were used as an initial model of RB1-Fab. Initial models of the 1A6 and 1D4 Fv molecules were generated using AbodyBuilder2 (https://doi.org/10.1038/s42003-023-04927-7, [38]. Accessed on 28 April 2023). Initial models for the 1A6 and 1D4 CH/CL domains were generated from identical entries within the PDB (PDB 1AJ7 and 1LIL, respectively). Initial Fab-Fab models of RB1-1A6 and RB1-1D4 complexes were generated by docking the RB1 Fab model along with AbodyBuilder2 models of the Fvs and CH/CL models from the PDB into the maps using Chimera software (version 1.13.1). All further model building was carried out in Coot software [39] (version 0.8.9.1) and involved manual placement and adjustment of backbone traces and side-chain positioning within the density map. Real-space refinement was carried out in Phenix software [40,41] (version 1.20.1). Structure quality was assessed by Ramachandran analysis, MolProbity clash scores [42], and map-to-model FSC.

### 2.5. In Silico Energetics Calculation Using Molecular Operating Environment Software

The MOE software (version 2022.02) calculated interaction energetics between Fab-Fab interface residues of 1A6-RB1 and 1D4-RB1 complexes obtained from cryo-EM structures. It considered van der Waals interactions, electrostatic interactions, hydrogen bonding, and solvation effects for stability assessment.

### 2.6. Structural Visualization

Pymol^TM^ (Schrödinger, Inc. New York, NY, USA) version 2.2.3 was used for all the structural visualization and later for the structural analyses and superposition of the RB1-RSVF crystal structure (6OUS) for the comparison in structural mimicry with each of the 1A6 or 1D4 to RB1 complexes.

### 2.7. Immunization Study with 1A6 Anti-ID

The animal experiments were approved by the Institutional Animal Care and Use Committee (IACUC), Merck & Co., Inc. (Rahway, NJ, USA), and conducted in compliance with our institution’s IACUC guidelines and the National Institutes of Health’s Guide for Care and Use of Laboratory Animals. Female BALB/c mice were obtained from Charles River Laboratories and housed in polycarbonate cages, with microisolator lids at MSD Research Laboratories (MRL), with access to standard rodent chow and water ad libitum, and daily health monitoring.

**Figure 1:**
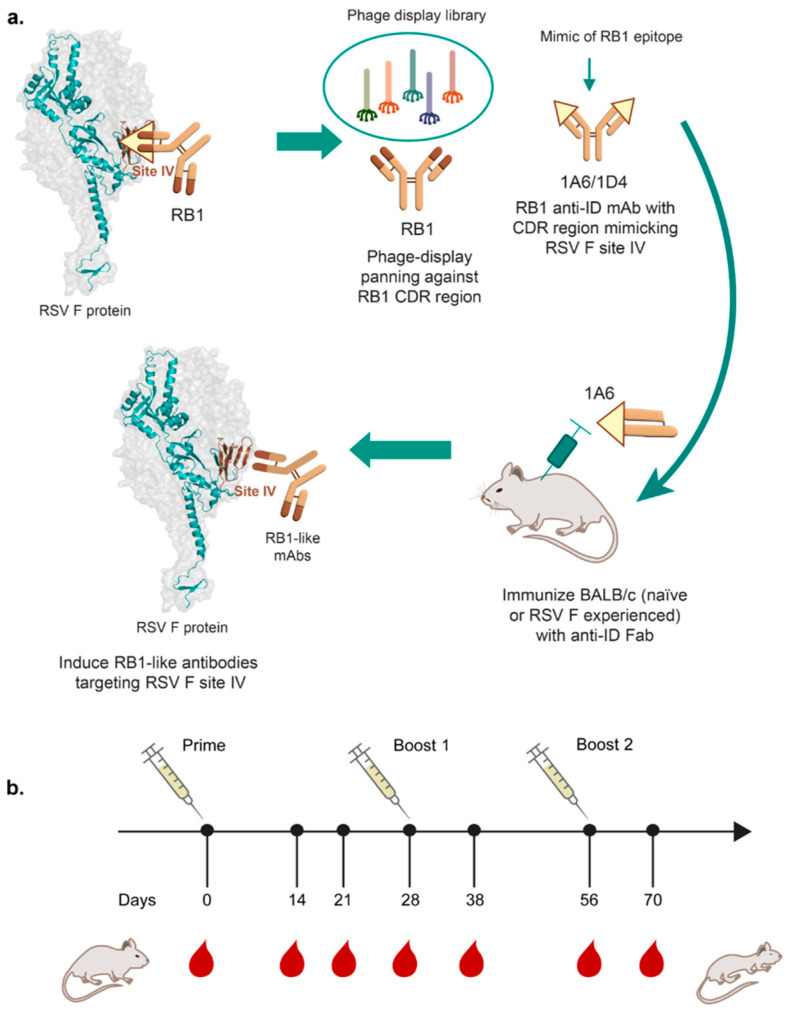
**Rationale and experimental design of the study in BALB/c mice.** (**a**) Rationale for using Anti-IDs for RSV F site-specific booster vaccine. The concept is illustrated with an Anti-RSV F site IV binding antibody RB1; (**b**) schematic describing this study’s experimental design.

This immunization assay was conducted to evaluate the immune response generated due to the use of anti-ID 1A6 Fab as a booster (Figure 1 and Table 1). There were five groups of female BALB/c mice (n = 8 in each group) that were intramuscularly immunized with one alum adjuvanted prime dose (on Day 0), followed by two boosters (on Days 28 and 56) formulated only in PBS. The immunization doses were kept at 100 µL with 50 µL injected into each of the quadriceps for each animal. The study included a positive control group receiving 2 µg RSV F as prime and for two boosters, and four test groups. Groups 1 and 2 received 2 µg RSV F as prime, with Group 2 additionally receiving 100 µg 1A6 Fab for two boosters. Groups 3, 4, and 5 received 10 µg, 50 µg, and 100 µg doses of 1A6 Fab as prime and one booster, followed by a final booster with 2 µg RSV F. The animals were bled via tail veins on Day 0 to obtain baseline titers, followed by a prime dose. Post prime, animals were similarly bled on Days 14 and 21 for testing. Post booster 1 bleed was collected on Day 38. Post booster 2 bleed was the terminal bleed and was performed on Day 70. For the terminal bleed procedure, mice were euthanized using CO_2_ asphyxiation (30% to 70% chamber volume inhaled/minute), followed by cardiac blood collection, as per institutional IACUC guidelines. The sera from all bleeds were tested for Anti-RSV F and Anti-Fab titers by ELISA.

#### 2.7.1. RSV F and Anti-Fab Capture ELISA

Anti-RSV F antibodies or Anti-Fab titers were determined using an RSV F (Evotec) or 1A6 IgG (GenScript, Piscataway, NJ, USA) capture ELISA at 1 µg/mL. The recombinant protein antigen was used as the coating antigen (i.e., RSV F or 1A6 IgG at 1 µg/mL) and incubated overnight at 4 °C using 96-well NUNC Maximmunosorp Plates or Immulon Plates (Thermo Fisher Scientific, Waltham, MA, USA). Before adding the diluted sera into the ELISA plates, the plates were washed with PBS-T (200 µL/well) using the Titertek M96 Washer. The assay plates were then blocked with Blocker™ Casein in PBS (Thermo Fisher Scientific, Waltham, MA, USA) at room temperature (RT) for 2 h. Similar washing steps were followed. For the Anti-RSV F capture ELISA, mouse sera were diluted to 1/450, followed by three-fold dilutions for 8 points, and incubated in the ELISA plate for 1 h at RT. The assay included an Anti-RSV F mouse serum as a positive control and an anti-SARS CoV2 mouse serum as a negative control. Next, the plates were incubated with 1:1000 HRP-conjugated goat anti-mouse IgG Fc (Jackson ImmunoResearch Inc., West Grove, PA, USA) for 1 h. The plates were then developed using the HRP TMB-substrate (Thermo Fisher Scientific, Waltham, MA, USA) for approximately 4 min at RT, and reaction was stopped using stop solution (TMB stop solution Thermo Fisher Scientific, Waltham, MA, USA). Finally, the plates were read at 450 nm using the SpectraMax Plus 384 microplate reader (Molecular Devices, San Jose, CA, USA) with SoftMax Pro 7.1 software. A similar procedure was followed for Anti-Fab ELISA, where the mouse sera were diluted from 1/50, followed by three-fold dilutions for 8 points. After a similar incubation as described above, the plates were washed and allowed to interact with 1:10,000 Peroxidase AffiniPure™Goat Anti-Human IgG, Fcγ fragment specific (Jackson ImmunoResearch Inc, West Grove, PA, USA). The plates were developed and then read.

#### 2.7.2. Competition Alpha-LISA to Test for RSV F Site IV-Specific Immune Response

Terminal bleed serum samples from Groups 1, 2, and 5 underwent competitive binding immunoassay to D25 or M2D2 antibody, binding to RSV F protein using AlphaLISA format. Serum samples were 3-fold serially diluted using a HiBlock buffer (Perkin Elmer, Waltham, MA, USA) in a 10-point titration, mixed with AlphaLISA acceptor beads conjugated to RSV F protein (100 μg/mL), and incubated for 30 min at room temperature. In this experiment, 10 μL of diluted samples were mixed with 5 μL of AlphaLISA acceptor beads conjugated to RSV F protein. All the following steps were carried out in the dark. 10 μL of biotinylated D25 (40 ng/mL) or M2D2 (2.5 ng/mL) antibody was added and allowed to incubate for 60 min, followed by 90 min of incubation with 25 μL streptavidin-coated donor AlphaLISA beads (20 μg/mL). The AlphaLISA signal was read on a PHERAStar FS (BMG Labtech, Ortenberg, Germany) plate reader (excitation/emission wavelengths at 680 nm/615 nm), and inhibitory antibody titers (IT50) were determined. Values were fit with a four-parameter fit in Graph Pad Prism 10.2.2, with samples hitting 50% competition or greater given the IT50 value of 50.

#### 2.7.3. GFP-RSV-A Long Virus Neutralization Assay

Mouse sera from groups 1 and 2 at days 14, 21, 38, and 70 were heat-inactivated and diluted in EMEM with Glutamine/2%FBS. Sera samples were diluted to an initial concentration of 1:10 and then titrated 3-fold for 8 points, and the RSV-A Long live virus was diluted at 1:100, all in EMEM/2%FBS (Cytiva, Marlborough, MA, USA) medium, in the presence of Pen/Strep antibiotics (Thermo Fisher Scientific, Waltham, MA, USA), on a 96-well U-bottom plate (Corning, Corning, NY, USA). Equal volumes of pre-diluted antibody and virus were combined on a new plate and then incubated at 37 °C for 1 h. HEp-2 cells were trypsinized, counted, and plated into a 96-well flat clear-bottom black polystyrene TC-treated microplate (Corning, Corning, NY, USA). The virus–antibody mixture was transferred into pre-seeded HEp-2 cell plates and incubated for 42–48 h at 37 °C with 5% CO_2_. After incubation, the plates were scanned using an Acuman^®^ Cellista (SPT Labtech, Melbourn, UK). Percent neutralization was calculated for each antibody dilution. Four-parameter curve fitting using GraphPad Prism 10.2.2 determined the NT50 value, thus evaluating neutralization activity against RSV-A Long live virus in mouse sera samples.

## 3. Results

### 3.1. 1A6 and 1D4 Fabs Bind to RB1 on Different Epitopes, and 1A6 Structurally Mimics the RSV F Binding to RB1

**Figure 2:**
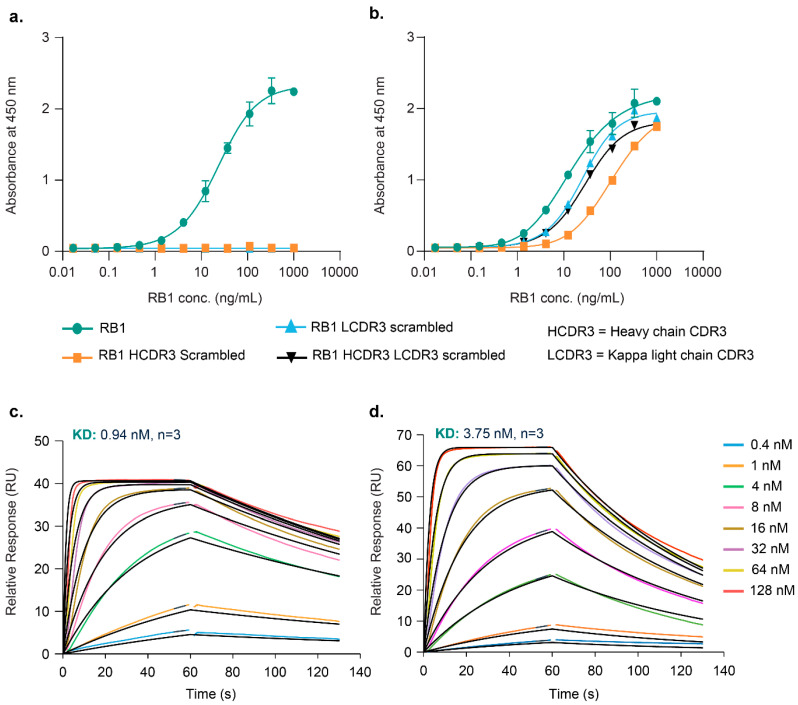
**RB1 interacts differently with 1A6 and 1D4 Fabs.** ELISA of HCDR3 and LCDR3 scrambled constructs of RB1 with 1A6 and 1D4 Fabs. (**a**) RB1-1A6 interaction by ELISA; (**b**) RB1-1D4 interaction by ELISA; (**c**,**d**) Biacore study for RB1 IgG interaction with 1A6 and 1D4 anti-ID Fabs, respectively. The black lines show the 1:1 fit for curves at several different 1A6 or 1D4 Fab concentrations ranging from 0.4 nM to 128 nM. Reported kinetic parameters are the averaged values from three runs.

Our previous work [26] with anti-idiotypic antibodies 1A6 and 1D4 demonstrated their binding to RB1, with 1A6-RB1 and 1D4-RB1 ELISA EC50s being 23 ng/mL and 14.4 ng/mL, respectively (Figure 2a,b), and the respective affinities estimated at 0.94 nM and 3.75 nM (Figure 2c,d and Table 2). Scrambling RB1 CDR3 regions showed that 1A6 interaction was abrogated, while 1D4 was more impacted by HCDR3 scrambling (117 ng/mL) than scrambling LCDR3 alone (24.5 ng/mL) or in combination with HCDR3 (28.3 ng/mL), which had similar effects, suggesting their equal impact (Figure 2b). These findings indicate RB1 has distinct epitopes for interaction with 1A6 and 1D4, with different affinities, which were further resolved by structural studies. This experiment highlighted the critical role of RB1 HCDR3 and LCDR3 in interaction with 1A6 and underscored the greater importance of RB1 HCDR3 in the 1D4 interaction.

**Figure 3:**
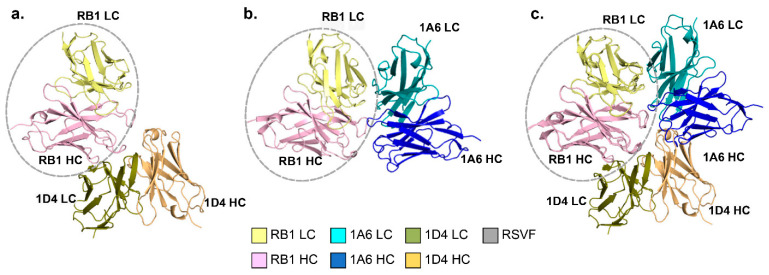
Cryo-EM structures of 1A6 and 1D4 anti-ID Fab light (LC) and heavy chain (HC) variable domains indicate different modes of interaction with RB1 Fab variable domains and superposition of the RB1-RSV F complex crystal structure on the two Fab complexes above, with RB1 variable domains circled in gray dashed lines. (**a**) RB1 HC (light pink) interacts with 1D4 LC (deep olive) and HC (beige); (**b**) RB1 HC (light pink) and LC (pale yellow) interact with 1A6 with both LC (cyan) and HC (blue); (**c**) RB1-1D4 and RB1-1A6 structural superposition showing different modes of binding with RB1 Fab.

**Figure 4:**
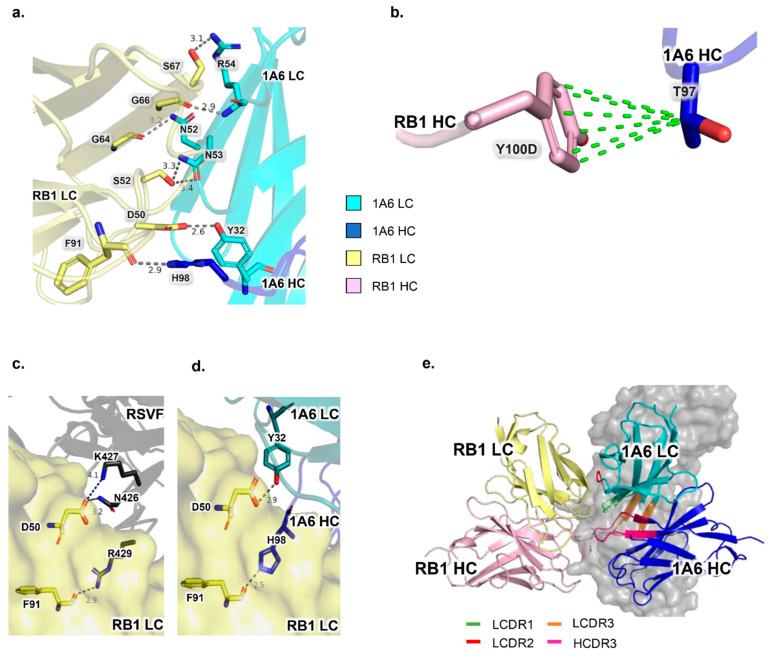
RB1 light chain (LC) and heavy chain (HC) interact via both chains of 1A6 anti-ID and 1A6 anti-ID mimics RSVF epitope in binding to RB1. RB1 Residues D50 and F91 interact with both RSV F and 1A6. (**a**) RB1 LC (pale yellow) interacts with 1A6 HC (blue) and LC (cyan) via hydrogen bonding (gray dotted lines), with the bond distances (in Å) estimated using PyMOL; (**b**) RB1 HCDR3 loop in HC (light pink) interacts with 1A6 HCDR3 loop in HC (blue) via hydrophobic interaction (green dotted lines); (**c**) site IV residues N426, K427, and R429 are important in interaction with RB1; (**d**) site IV RSV F interaction mimicry was shown by 1A6 LC and HC residues Y32 and H98, respectively. (**e**) Superimposition of RB1-RSV F crystal structure (PDB 6OUS), where RSV F monomer is shown in surface representation on RB1-1A6 structure. The CDR loops in 1A6 that are taking part in the interaction with RB1 are shown in different colors (LCDR1, LCDR2, LCDR3, and HCDR3). The 1A6 light and heavy chains are shown to occupy the surface density of the RB1 binding site of RSV F. Note: Colors represent atom types (oxygen in red, nitrogen in blue). Carbon atoms are colored by chain. Dashes represent hydrogen bonds with distances annotated.

To further understand the atomic interaction between RB1 and the anti-IDs, we performed Cryo-EM studies on the complexes. These studies confirmed that 1A6 and RB1 interaction involves both heavy and light chains (Figure 3b,c, Table 2 and Supplementary Figure S1), as observed by the scrambled CDR interaction. All significant interactions between 1A6 and RB1 are through hydrogen bonding, except for one hydrophobic interaction (Figure 4a,b). Moreover, the interacting residues were found both in the CDR and framework regions (FR). In the interaction between 1A6 LC and RB1 LC, the residues N53 and R54 (LFR3) on 1A6 form a network of hydrogen bonding with the G66, S52, and S67 residues (LFR3) on RB1 (Figure 4a). Additionally, the amine group of the amide side chain of N52 on 1A6 (LCDR2) forms a hydrogen bond with the backbone carboxyl group of G64 (LFR3) on RB1 (Figure 4a). The 1A6 Y32 (LCDR1) hydroxyl group of the side chain hydrogen bonds with the carboxyl group of the side chain of RB1 D50 (LCDR2) (Figure 4a). In the interaction between 1A6 HC and RB1 LC, the 1A6 H98 (HCDR3) imidazole side chain interaction with the RB1 F91 (LCDR3) backbone carbonyl group is also stabilized by a hydrogen bond (Figure 4a). In the interaction between 1A6 HC and RB1 HC, 1A6 T97 (HCDR3) shows hydrophobic interaction with the beta carbon of the aliphatic side chain with the aromatic ring of RB1 Y100D (HCDR3) (Figure 4b), via a CH-π interaction. Interestingly, although 1D4 anti-ID interacts with RB1 via both the variable heavy and light chains, they only engage the heavy chain of RB1 (Figure 3a,c, Supplementary Figure S5, Supplementary Tables S1 and S2). This structural observation and the scrambled CDR3 interaction data results delineate the importance of the RB1 heavy chain in interacting with 1D4 heavy and light chains.

Our previous work showed that RB1 can no longer bind to the RSV F trimer if pre-bound to 1A6, whereas the 1D4-RB1 complex can still show some binding to RSV F [26]. To investigate this observation further, we used Cryo-EM to confirm that these antibodies bind to RB1 via different modes. In the interaction between RB1 and 1A6 or RSV F, the residues F91 on LCDR3 and D50 on LCDR2 of RB1 were found to interact with RSV F and 1A6 (Figure 4c,d, Table 3 and Table 4). Specifically, D50 on RB1 forms a salt bridge with the K427 side chain amine and a hydrogen bond with the amine group of the amide side chain of N426 of RSV F (Figure 3c, Table 3 and Table 4). This interaction was mimicked by 1A6 by forming a hydrogen bond with Y32 (LCDR1) of 1A6 to D50 (LCDR2) on RB1, as mentioned before (Figure 4d, Table 3, Table 4). Additionally, the F91 residue on RB1 interacts via its backbone carbonyl group with the side chain amine of R429 on RSV F (Figure 4c). A similar interaction involving the histidine imidazole side chain of residue H98 on the HCDR3 of 1A6 with the F91 residue on RB1 was also observed (Figure 4d, Table 3). There was no commonality in the interaction of RSV F-RB1 with 1D4-RB1 (Supplementary Figure S5, Supplementary Table S2). Additionally, upon superimposition of the RSV F-RB1 crystal structure on the 1A6-RB1 cryo-EM structure, we found that the buried surface area between the monomer of RSV F site IV and RB1 is 1149 Å^2^, whereas that of the 1A6-RB1 complex is 1405 Å^2^. Since 1A6 is found to bind RB1 at the same site as RSV F site IV, 1A6 spans the entire footprint of the RB1 epitope on RSV F (Figure 4e). Based on these studies, 1A6 was chosen for our mouse immunization studies to test its ability to induce site IV binding anti-RSV F antibodies.

### 3.2. 1A6 Functionally Mimics the RSV F Site IV

**Figure 5:**
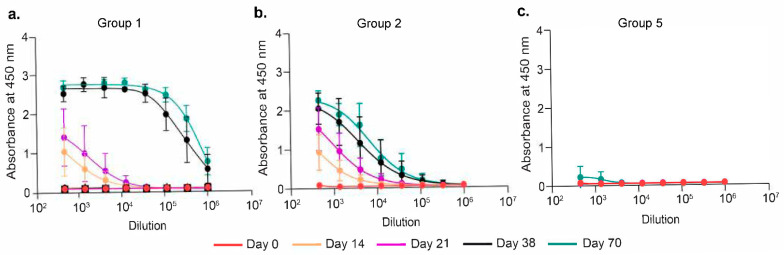
**Anti-IDs Boost Anti-RSV F Response.** Anti-RSV F capture ELISA shows different titer boosts across various groups. (**a**) Group 1 animals were primed and boosted twice with 2 µg RSV F (RSV F ×3); (**b**) Group 2 animals were primed with 2 µg RSV F and boosted twice with 100 µg 1A6 Fab (RSV F-1A6 ×2); (**c**) Group 5 animals were primed and boosted once with 1A6 Fab, followed by a booster with 2 µg RSV F (1A6 ×2-RSV F).

To test if 1A6 Fab could elicit RSV F site IV-directed humoral responses, we primed Balb/c mice (eight per group) and boosted them one month apart with the antigens, as shown in Table 1. Group 1 animals were primed with RSV F in alum and subsequently boosted with RSV F at Days 28 and 56 in PBS; group 2 mice were primed with RSV F in alum and boosted at Days 28 and 56 with 1A6 in alum; and Groups 3, 4, and 5 were primed with different concentrations (10 µg, 50 µg, and 100 µg) of 1A6 Fab in alum and boosted with 1A6 Fab in PBS at Day 28, followed by the final boost with 2 µg of RSV F in PBS at Day 56. All mice were bled every two weeks to assay for their humoral immune response (Figure 1 and Table 1). On Day 21, both Group 1 (RSV F ×3) and Group 2 (RSV F-1A6 ×2) animals exhibited similar anti-RSV F titers, which was expected as both groups received a single immunization with RSV F in alum. In Group 1, the first boost shot induced a robust anti-RSV F immune response at Day 38. In contrast, the second shot did not show much enhancement in anti-RSV F titers in these mice (Figure 5a). In Group 2 animals, the first boost with 1A6 Fab increased the anti-RSV F titers, which were further enhanced following the second boost shot, indicating that this anti-ID mAb Fab was able to improve the site IV-specific response elicited by RSV F priming (Figure 5b). There are minimal anti-RSV F titers seen in animals in Groups 3–5, even following the RSV F boost shot (Figure 5c, Supplementary Figure S6a,b). The lack of anti-RSV F response was not due to the failure of immunization since there are Anti-Fab titers against the immunogen which were significantly boosted after the second shot of 1A6 Fab (Supplementary Figure S7c–e). Similarly, Group 2 animals showed Anti-Fab titers two weeks post boost 1, which increased after the second booster (Supplementary Figure S7b). These data indicate that 1A6 Fab can only be used to boost site IV-specific response and cannot be used as a prime immunogen.

**Figure 6:**
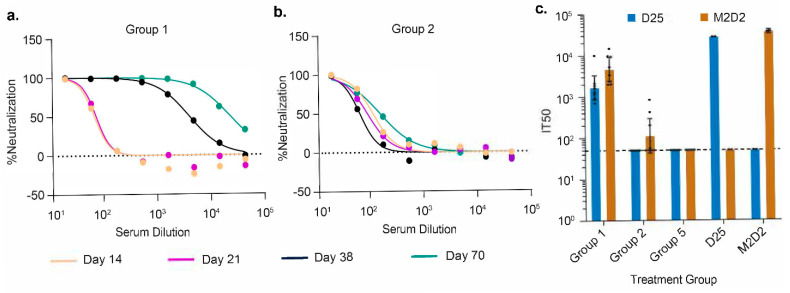
**Anti-IDs boost Anti-RSV neutralization titer and show site IV specificity.** (**a**,**b**) Anti-RSV titer boost for pulled sera from animals of Group 1 (RSV F ×3) and Group 2 (RSV F-1A6 ×2), respectively, from different bleeds. The dotted line indicates the limit of detection for the experiment. (**c**) Anti-RSV F site Ø and site IV titer boost from terminal bleed sera from animals of treatment Groups 1, 2, and 5 (1A6 ×2-RSV F), respectively. The IT50 for each animal is plotted, and the dotted line at IT50  =  50 indicates the assay’s limit of detection (LOD). Animals with no competing antibody titers were assigned a titer at the LOD. M2D2 (in red) and D25 (in blue) were used to test RSV F site IV and site Ø competition, respectively.

In the RSV neutralization assay, we tested the functional activities of the serum antibodies from animals in Groups 1 and 2. Group 1 (RSV F ×3) animals showed a robust increase in neutralization titers from prime to first boost and second boost in pooled sera (Figure 6a and Table 5), as well as strong neutralization titers for individual animals (Supplementary Figure S8a and Supplementary Table S3). Group 2 (RSV F-1A6 ×2) animals, when tested for neutralization titers from pooled sera, demonstrated a 2.4-fold increase in neutralizing activity from Day 38 to Day 70 post boost 2 (Figure 6b and Table 5). This indicates that 1A6 Fab was working as a booster antigen vaccine in eliciting more RSV-neutralizing antibodies. Interestingly, there was a decline in neutralizing activity from Day 21 to 38, indicating the loss of RSV F-specific response by Day 38 due to the natural decay of the antibodies in the tested animals (Figure 6b). When analyzing the individual animals, it was found that animals 11, 13, and 14 from Group 2 exhibited positive neutralization titers (Supplementary Table S3), with an overall mean IC50 (dilution factor) of 152.2, which agrees with the pooled sera IC50 (dilution factor) of 159 (Supplementary Table S3 and Table 5).

To determine the RSV F site specificity of the boosted response of the animal sera in Groups 1, 2, and 5, we terminally bled these mice at the end of the study on Day 70 and used this bleed for alpha-LISA competition assays. RSV F Site Ø, being immunodominant, was tested as a control, using the D25 [17] antibody, whereas the site IV competition was tested using an RSV F site-IV specific antibody, M2D2; details of this antibody were described before [43]. In Group 1 (RSV F ×3) animals, the inhibition titer for both D25 and M2D2 was above the limit of detection, suggesting the presence of neutralizing antibodies against site Ø and site IV (Figure 6c). This finding indicates that the animals in Group 1, immunized with RSV-F, generated a diverse immune response targeting multiple sites of RSV F. In contrast, in Group 2 animals (RSV F-1A6 ×2), the inhibition titer for M2D2 was above the detection limit in three animals (10, 11, and 13, in increasing order), while no D25 competition was observed. These results indicate that animals primed with RSV F enhanced their immune response by targeting site IV memory B cells due to the 1A6 Fab booster. Group 5 (1A6 ×2-RSV F) animals showed no D25 or M2D2 specific antibodies, corroborating the observation of no RSV F titer on capture ELISA, suggesting 1A6 anti-ID is not a good choice for prime immunization.

## 4. Discussion

Our structural analyses have provided compelling evidence to support our initial hypothesis regarding an anti-ID that mimics the RB1 epitope on RSV F site IV. Octet competition data from our previous work [26] have further corroborated these results. Previous data demonstrated that when the RB1 epitope is bound by 1A6, the binding of RSV F is fully inhibited, indicating complete competition. Conversely, 1D4 only shows partial competition with RSV F in binding to RB1 [26]. Our cryo-EM data for the two complexes, when superimposed to the RB1-RSV preF crystal structure [23], showed that while 1A6 shares the same binding site as RSV F to RB1, 1D4 only partially blocks RSV F from binding to RB1. In conclusion, our Cryo-EM study explains the different binding modes of 1A6 and 1D4 to RB1, which aligns with the observations from the earlier octet studies [26].

Superimposition of the crystal structure of RB1-RSV preF and the cryo-EM structure of 1A6-RB1 unambiguously illustrated that 1A6 mimicked RSV F in binding to RB1. Furthermore, we have previously shown, using crystal structure, that RSV F residues 426–429 and 432 are important, and an alanine scanning experiment revealed that residues 429 and 432 are crucial for the interaction with RB1 [23]. Our structural study reveals that RSV F residues N426 and K427 are structurally mimicked by Y32 of 1A6. Similarly, we observed structural mimicry between R429 on RSV F and H98 of 1A6. Although the exact residues in RSV F and 1A6 Fab involved in the interaction with RB1 are different, a common interaction pattern is observed. For instance, the hydrogen bonding interaction between N426 of RSV F with the D50 side chain on RB1 LC is mimicked by a hydrogen bond formed by the 1A6 LC Y32 side chain with the RB1 LC D50 side chain. Similarly, the critical residue R429 on RSV F, as identified in our previous study [23], forms a hydrogen bond with F91 of the RB1 LC and is structurally paralleled by a hydrogen bond formed by the H98 side chain of 1A6 HC with F91 of RB1 LC. These findings solidify our assertion that 1A6 is an excellent structural mimic of the RB1 epitope on RSV F. Site IV comprises residues 422 to 468 [23]. The RSV F-RB1 crystal structure revealed that the RB1 epitope is between residues 426 and 447 [23]. The 1A6 Fab specifically mimics the RB1 epitope on RSV F. Since we found that the 1A6 footprint spans the RB1 binding site on RSV F, this further strengthens the evidence that 1A6 is an excellent structural mimic of RSV F site IV.

The in vivo animal experiments were carried out to provide immunological proof of principle of being able to boost epitope-specific immune responses. Furthermore, since the RSV F site Ø is an immunodominant site [44] and is also prone to mutation, we propose that in the event of developing resistance, shifting the immune response towards a different highly conserved RSV F site [23,24,25,45] would be more prudent. Site IV is more conserved than Site Ø, and in particular, the RB1 binding site has 99.8% sequence identity observed among the 15,527 available RSV F sequences in GenBank as of April 2024 (internal analyses). Therefore, we targeted the epitope of RB1, within the site IV of RSV F, by raising anti-idiotypic antibodies against RB1. Interestingly, a pre-print shows that a single L305I mutation on RSV A F results in a decrease in neutralization potency of site Ø antibodies by ~4x, while that of the site IV antibodies is unaffected [46]. Furthermore, recent research also demonstrated the importance of anti-idiotypic antibodies for targeting RSV-neutralizing B cell receptors [47]. Therefore, this study is a hypothesis-builder for a future epitope or site-specific vaccination, shifting the response to avoid immunodominant and original antigenic sin pitfalls. To evaluate 1A6 as an internal image of an RB1-binding epitope on RSV F, we performed studies to test its ability to elicit antibodies against the RSV F site IV. The results showed that Group 2 (RSV F-1A6 ×2) mice, primed with RSV F and boosted with 1A6 Fab, exhibited considerably lower Anti-RSV F titers than the Group 1 (RSV F ×3) control group. Notably, the RSV F protein comprises hundreds of antigenic epitopes, while 1A6 mimics only one, explaining the weaker antibody response in Group 2. However, the observed increase in the Anti-RSV F titer following the initial boost with 1A6 Fab indicates successful activation and amplification of very low-frequency RSV F-specific memory B-cells targeting the RB1 epitope, potentially mimicked by 1A6 as RSV F site IV. Moreover, the boost in Anti-1A6 Fab titers from prime to boost demonstrates the successful immunogenicity of human 1A6 Fab. Our findings also highlight that 1A6 Fab can only function as a booster antigen, not a prime immunogen, to trigger the RB1-epitope-specific antibody response.

Further analysis of the pooled animal sera revealed an approximately 2.4-fold increase in the live RSV-A neutralization titer between the two boosts in Group 2 (RSV F-1A6 ×2) animals. This increase in neutralization titer strongly supports 1A6 as a surrogate epitope-specific antigen in boosting RSV F site IV antibody response. The alpha-LISA competition assay with a site IV mAb on the terminal bleeds demonstrated that two of the three animals in Group 2 (RSV F-1A6 ×2) had increased neutralizing titers competing with site IV-specific antibodies. These results provide the first proof of immunological principle for epitope-specific boosting of humoral response with anti-ID antibodies. When considering anti-ID Fabs as a surrogate RSV-boosting vaccine, the antibody titer boost was modest, and there is ample room for improvement in anti-RSV F neutralization titers. Future studies will be directed towards increasing the amplitude of the epitope-specific immune responses using the anti-ID as an immunogen.

In our experience, when RSV F absorbed to alum is used as an antigen, it reaches a steady-state serum antibody titer by 35–42 days. This is in line with the kinetics of B cell responses (with a peak at around 3 weeks) and the average half-life of IgG being 21 days [48,49,50,51]. Furthermore, the subunit antigens are cleared very quickly (less than a week) after immunization. Hence, it is very unlikely for the anti-RSV F response to keep increasing beyond Day 42 until Day 70. Additionally, we would like to point out that there was a drop in the neutralization titer from Day 21 to Day 38 (Day 21 IC50 = 92.5 and Day 38 IC50 = 66.8 dilution factors). This neutralization titer at Day 38 was further boosted after the second anti-ID boosting at Day 56 to an IC50 of 159 at Day 70, resulting in a 2.4-fold boost over the Day 38 titer. Additionally, in Figure 6c, at Day 70 there was an increase in site IV competitive ELISA titers with no increase for site Ø competitive titers. These observations give us strong confidence that the serum titer increase for site IV antibody is due to 1A6 acting as a booster antigen.

## 5. Conclusions

In conclusion, this study gives us encouragement that this immunological principle can go further, beyond a single epitope, by displaying multiple RSV epitopes on nanoparticle platforms, where the anti-IDs have the potential to be used as booster vaccines to elicit desired epitope-specific antibody responses. Needless to say, this concept can be adapted for other viruses (HIV, HMPV, etc.) beyond RSV. The use of strong adjuvants like AS01 for the elderly and TLR8/9 adjuvants for neonates will be needed to further increase the amplitude of immune responses in the elderly and neonates, respectively. These types of experimental evaluations are planned for post-optimization of the anti-ID antigen display and delivery but beyond the scope of this current manuscript. The current level of immune responses is insufficient to carry out viral challenge studies in animals. This proof-of-principle study we show here paves the way for further (aforementioned) studies in order to achieve protective immunity in risk populations.

## Figures and Tables

**Table 1:** BALB/c immunization plan.

Groups	Immunization Doses	Day 0 (with Alum)	Day 28 (with PBS)	Day 56 (with PBS)
1	RSV F ×3	2 µg RSV F	2 µg RSV F	2 µg RSV F
2	RSV F-1A6 ×2	2 µg RSV F	100 µg 1A6	100 µg 1A6
3	1A6 ×2-RSV F	10 µg 1A6	10 µg 1A6	2 µg RSV F
4		50 µg 1A6	50 µg 1A6	2 µg RSV F
5		100 µg 1A6	100 µg 1A6	2 µg RSV F

**Table 2:** Biacore determination of the kinetics of binding of Anti-IDs to RB1.

**RB1**	**1A6 Fab**			**1D4 Fab**		
	***k*** **_on_** ** (M^−1^s^−1^)**	***k*** **_off_** ** (s^−1^)**	***K*** **_D_** ** (nM)**	***k*** **_on_** ** (M^−1^s^−1^)**	***k*** **_off_** ** (s^−1^)**	***K*** **_D_** ** (nM)**
	6.67 × 10^6^	6.28 × 10^−3^	0.94	3.50 × 10^6^	1.30 ×10^−2^	3.75

*K*_D_—affinity or equilibrium dissociation constant, *k*_on_—association rate, *k*_off_—dissociation rate, M—molar, nM—nanomolar, s—seconds.

**Table 3:** 1A6 interaction with RB1.

1A6 Site	Residue	RB1 Site	Residue	ΔG (kcal/mole)
LCDR1	Y32	LCDR2	D50	−2.2
LCDR2	N52	LFR3	G64	−1.7
LFR3	N53		S52	−1.5
	R54		G66	−5.6
	R54		S67	−1.9
HCDR3	H98	LCDR3	F91	−2.2
	T97	HCDR3	Y100D	−0.5

ΔG calculations were performed on MOE; LCDR = VL CDR region, HCDR = VH CDR region, LFR = VL framework region.

**Table 4:** ΔG of interaction with residues F91 and D50 of RB1 with corresponding binding partner residues of 1A6 anti-ID or RSV F.

Chain A	Site	Residue	Chain B	Site	Residue	ΔG (kcal/mole)
RSV F	IV	N426	RB1	LCDR2	D50	−3.5
		K427				−2.88
		R429		LCDR3	F91	−4.2
1A6	LCDR1	Y32	RB1	LCDR2	D50	−2.2
	HCDR3	H98		LCDR3	F91	−2.2

ΔG calculations were performed on MOE; LCDR = VL CDR region, and HCDR = VH CDR region.

**Table 5:** Pooled sera IC50 and neutralization titer fold change between boosts.

Pooled Sera Samples	RSV F ×3		RSV F-1A6 ×2	
Bleeds	Day 38	Day 70	Day 38	Day 70
IC50 (dilution)	4040	23,215	67	159
Fold change between Booster 1 and 2	5.7		2.4	

## Data Availability

The data sets generated and/or analyzed during the current study are available in the article or Supplementary Materials or from the corresponding author on reasonable request. The refined 1A6-RB1 and 1D4-RB1 structures and corresponding cryo-EM maps have been deposited within the Protein Data Bank (PDB) and the Electron Microscopy Data Bank (EMDB) under accession codes 9MML and EMD-48393 (1A6-RB1) and 9MMV and EMD-48404 (1D4-RB1).

## Supplementary Materials

The following supporting information can be downloaded at: https://www.mdpi.com/article/10.3390/vaccines13010035/s1, Figure S1: Protein sample quality assessment for Cryo-EM; Figure S2: Fab-Fab particles Observed In the Field of View; Figure S3: 1A6-RB1 complex cryo-EM data processing; Figure S4. 1D4-RB1 complex cryo-EM data processing; Figure S5: 1D4 Anti-ID Light chain (LC) and Heavy chain (HC) interact only with RB1 HC; Figure S6. Anti-RSV F titer boost for 1A6 ×2-RSV F groups that were primed with different doses of 1A6 Fab; Figure S7. Mean Anti-Fab titer boost for animals from Groups 1–5 on different bleeds. Figure S8. Anti-IDs Boost Anti-RSV Neutralization Titer; Table S1: Cryo-EM data collection, refinement, validation, and statistics; Table S2: 1D4 interaction with RB1. Table S3: Individual animal terminal bleed (Day 70) sera IC50.

## Abbreviations

Anti-ID antibodies	Anti-idiotypic antibodies
HC	Heavy chain
LC	Light chain
CDR	Complementary determining region
RSV	Respiratory Syncytial Virus
RSV F	Respiratory Syncytial Virus Fusion protein
Cryo-EM	Cryo-electron microscopy
SEC	Size exclusion chromatography

## References

[1] Jerne N.K., Roland J., Cazenave P.A. (1982). Recurrent idiotopes and internal images. EMBO J..

[2] Jerne N.K. (1974). Towards a network theory of the immune system. Ann. Immunol..

[3] Perelson A.S. (1989). Immune network theory. Immunol. Rev..

[4] Zhou E.-M., Lin M., Shoenfeld Y., Kennedy R.C., Ferrone S. (1997). Anti-Idiotype to Bluetongue Virus VP7 Antigen: Potential Diagnostic Reagent and Vaccine. Idiotypes in Medicine: Autoimmunity, Infection and Cancer.

[5] Kohler H., Pashov A., Kieber-Emmons T. (2019). The Promise of Anti-idiotype Revisited. Front. Immunol..

[6] Kieber-Emmons T., Monzavi-Karbassi B., Pashov A., Saha S., Murali R., Kohler H. (2012). The promise of the anti-idiotype concept. Front. Oncol..

[7] Huang W.L., Chuang S.C., Yang C.D. (2019). Anti-Idiotype Vaccine Provides Protective Immunity Against Vibrio Harveyi in Grouper (*Epinephelus coioides*). Vaccines.

[8] Naveed A., Rahman S.U., Arshad M.I., Aslam B. (2018). Immune Modulatory Potential of Anti-idiotype Antibodies as a Surrogate of Foot-and-Mouth Disease Virus Antigen. mSphere.

[9] Bancroft T., DeBuysscher B.L., Weidle C., Schwartz A., Wall A., Gray M.D., Feng J., Steach H.R., Fitzpatrick K.S., Gewe M.M. (2019). Detection and activation of HIV broadly neutralizing antibody precursor B cells using anti-idiotypes. J. Exp. Med..

[10] Siebert N., Eger C., Seidel D., Juttner M., Lode H.N. (2014). Validated detection of human anti-chimeric immune responses in serum of neuroblastoma patients treated with ch14.18/CHO. J. Immunol. Methods.

[11] Perez A., Mier E.S., Vispo N.S., Vazquez A.M., Perez Rodriguez R. (2002). A monoclonal antibody against NeuGc-containing gangliosides contains a regulatory idiotope involved in the interaction with B and T cells. Mol. Immunol..

[12] Rogovik A.L., Carleton B., Solimano A., Goldman R.D. (2010). Palivizumab for the prevention of respiratory syncytial virus infection. Can. Fam. Physician.

[13] Jones J.M., Fleming-Dutra K.E., Prill M.M., Roper L.E., Brooks O., Sanchez P.J., Kotton C.N., Mahon B.E., Meyer S., Long S.S. (2023). Use of Nirsevimab for the Prevention of Respiratory Syncytial Virus Disease Among Infants and Young Children: Recommendations of the Advisory Committee on Immunization Practices—United States, 2023. MMWR Morb. Mortal. Wkly. Rep..

[14] Ison M.G., Papi A., Athan E., Feldman R.G., Langley J.M., Lee D.-G., Leroux-Roels I., Martinon-Torres F., Schwarz T.F., van Zyl-Smit R.N. (2024). Efficacy and Safety of Respiratory Syncytial Virus (RSV) Prefusion F Protein Vaccine (RSVPreF3 OA) in Older Adults Over 2 RSV Seasons. Clin. Infect. Dis..

[15] Wroblewski D., Brust-Sisti L.A., Bridgeman M., Bridgeman M.B. (2024). Vaccines for Respiratory Syncytial Virus Prevention in Older Adults. Ann. Pharmacother..

[16] Wilson E., Goswami J., Baqui A.H., Doreski P.A., Perez-Marc G., Zaman K., Monroy J., Duncan C.J.A., Ujiie M., Ramet M. (2023). Efficacy and Safety of an mRNA-Based RSV PreF Vaccine in Older Adults. N. Engl. J. Med..

[17] McLellan J.S., Chen M., Leung S., Graepel K.W., Du X., Yang Y., Zhou T., Baxa U., Yasuda E., Beaumont T. (2013). Structure of RSV fusion glycoprotein trimer bound to a prefusion-specific neutralizing antibody. Science.

[18] Magro M., Andreu D., Gomez-Puertas P., Melero J.A., Palomo C. (2010). Neutralization of human respiratory syncytial virus infectivity by antibodies and low-molecular-weight compounds targeted against the fusion glycoprotein. J. Virol..

[19] Taylor G., Stott E.J., Furze J., Ford J., Sopp P. (1992). Protective epitopes on the fusion protein of respiratory syncytial virus recognized by murine and bovine monoclonal antibodies. J. Gen. Virol..

[20] Calder L.J., Gonzalez-Reyes L., Garcia-Barreno B., Wharton S.A., Skehel J.J., Wiley D.C., Melero J.A. (2000). Electron microscopy of the human respiratory syncytial virus fusion protein and complexes that it forms with monoclonal antibodies. Virology.

[21] Gilman M.S., Castellanos C.A., Chen M., Ngwuta J.O., Goodwin E., Moin S.M., Mas V., Melero J.A., Wright P.F., Graham B.S. (2016). Rapid profiling of RSV antibody repertoires from the memory B cells of naturally infected adult donors. Sci. Immunol..

[22] Graham B.S. (2019). Immunological goals for respiratory syncytial virus vaccine development. Curr. Opin. Immunol..

[23] Tang A., Chen Z., Cox K.S., Su H.P., Callahan C., Fridman A., Zhang L., Patel S.B., Cejas P.J., Swoyer R. (2019). A potent broadly neutralizing human RSV antibody targets conserved site IV of the fusion glycoprotein. Nat. Commun..

[24] Hause A.M., Henke D.M., Avadhanula V., Shaw C.A., Tapia L.I., Piedra P.A. (2017). Sequence variability of the respiratory syncytial virus (RSV) fusion gene among contemporary and historical genotypes of RSV/A and RSV/B. PLoS ONE.

[25] Mas V., Nair H., Campbell H., Melero J.A., Williams T.C. (2018). Antigenic and sequence variability of the human respiratory syncytial virus F glycoprotein compared to related viruses in a comprehensive dataset. Vaccine.

[26] Li A., Swanson M., Sullivan N., Homan Y., Nahas D., Mukhopadhyay S., Li H.H., Cao Y., Xu W., Tang H. (2023). Phage-derived anti-idiotype and anti-YTE antibodies in development of MK-1654 pharmacokinetic and immune response assays. Bioanalysis.

[27] Ban N., Escobar C., Garcia R., Hasel K., Day J., Greenwood A., McPherson A. (1994). Crystal structure of an idiotype-anti-idiotype Fab complex. Proc. Natl. Acad. Sci. USA.

[28] Cowton V.M., Owsianka A.M., Fadda V., Ortega-Prieto A.M., Cole S.J., Potter J.A., Skelton J.K., Jeffrey N., Di Lorenzo C., Dorner M. (2021). Development of a structural epitope mimic: An idiotypic approach to HCV vaccine design. NPJ Vaccines.

[29] Seydoux E., Wan Y.H., Feng J., Wall A., Aljedani S., Homad L.J., MacCamy A.J., Weidle C., Gray M.D., Brumage L. (2021). Development of a VRC01-class germline targeting immunogen derived from anti-idiotypic antibodies. Cell Rep..

[30] Olia A.S., Prabhakaran M., Harris D.R., Cheung C.S.-F., Gillespie R.A., Gorman J., Hoover A., Morano N.C., Ourahmane A., Srikanth A. (2024). Anti-idiotype isolation of a broad and potent influenza A virus-neutralizing human antibody. Front. Immunol..

[31] McLellan Jason S., Yang Y., Graham Barney S., Kwong Peter D. (2011). Structure of Respiratory Syncytial Virus Fusion Glycoprotein in the Postfusion Conformation Reveals Preservation of Neutralizing Epitopes. J. Virol..

[32] Myszka D.G. (1997). Kinetic analysis of macromolecular interactions using surface plasmon resonance biosensors. Curr. Opin. Biotechnol..

[33] Punjani A., Rubinstein J.L., Fleet D.J., Brubaker M.A. (2017). cryoSPARC: Algorithms for rapid unsupervised cryo-EM structure determination. Nat. Methods.

[34] Zheng S.Q., Palovcak E., Armache J.-P., Verba K.A., Cheng Y., Agard D.A. (2017). MotionCor2: Anisotropic correction of beam-induced motion for improved cryo-electron microscopy. Nat. Methods.

[35] Rohou A., Grigorieff N. (2015). CTFFIND4: Fast and accurate defocus estimation from electron micrographs. J. Struct. Biol..

[36] Punjani A., Fleet D.J. (2021). 3D variability analysis: Resolving continuous flexibility and discrete heterogeneity from single particle cryo-EM. J. Struct. Biol..

[37] He J., Li T., Huang S.-Y. (2023). Improvement of cryo-EM maps by simultaneous local and non-local deep learning. Nat. Commun..

[38] Abanades B., Wong W.K., Boyles F., Georges G., Bujotzek A., Deane C.M. (2023). ImmuneBuilder: Deep-Learning models for predicting the structures of immune proteins. Commun. Biol..

[39] Emsley P., Lohkamp B., Scott W.G., Cowtan K. (2010). Features and development of Coot. Acta Crystallogr. D Biol. Crystallogr..

[40] Adams P.D., Afonine P.V., Bunkóczi G., Chen V.B., Davis I.W., Echols N., Headd J.J., Hung L.W., Kapral G.J., Grosse-Kunstleve R.W. (2010). PHENIX: A comprehensive Python-based system for macromolecular structure solution. Acta Crystallogr. D Biol. Crystallogr..

[41] Afonine P.V., Poon B.K., Read R.J., Sobolev O.V., Terwilliger T.C., Urzhumtsev A., Adams P.D. (2018). Real-space refinement in PHENIX for cryo-EM and crystallography. Acta Crystallogr. D Struct. Biol..

[42] Chen V.B., Arendall W.B., Headd J.J., Keedy D.A., Immormino R.M., Kapral G.J., Murray L.W., Richardson J.S., Richardson D.C. (2010). MolProbity: All-atom structure validation for macromolecular crystallography. Acta Crystallogr. D Biol. Crystallogr..

[43] Xiao X., Tang A., Cox K.S., Wen Z., Callahan C., Sullivan N.L., Nahas D.D., Cosmi S., Galli J.D., Minnier M. (2019). Characterization of potent RSV neutralizing antibodies isolated from human memory B cells and identification of diverse RSV/hMPV cross-neutralizing epitopes. mAbs.

[44] McLellan J.S., Chen M., Joyce M.G., Sastry M., Stewart-Jones G.B., Yang Y., Zhang B., Chen L., Srivatsan S., Zheng A. (2013). Structure-based design of a fusion glycoprotein vaccine for respiratory syncytial virus. Science.

[45] Bin L., Liu H., Tabor D.E., Tovchigrechko A., Qi Y., Ruzin A., Esser M.T., Jin H. (2019). Emergence of new antigenic epitopes in the glycoproteins of human respiratory syncytial virus collected from a US surveillance study, 2015–2017. Sci. Rep..

[46] Oraby A., Stojic A., Elawar F., Bilawchuk L., Erwin K., Granoski M., Griffiths C., Arutyunova E., Lemieux M.J., Frederick W. (2024). A Single Amino Acid Mutation Alters the Neutralization Epitopes in the Respiratory Syncytial Virus Fusion Glycoprotein.

[47] Scharffenberger S.C., Wan Y.-H., Homad L.J., Kher G., Haynes A.M., Poudel B., Sinha I.R., Aldridge N., Pai A., Bibby M. (2024). Targeting RSV-neutralizing B cell receptors with anti-idiotypic antibodies. Cell Rep..

[48] Kobashi Y., Shimazu Y., Kawamura T., Nishikawa Y., Omata F., Kaneko Y., Kodama T., Tsubokura M. (2022). Peak IgG antibody titers against SARS-CoV-2 spike protein following immunization with the Pfizer/BioNTech BNT162b2 vaccine. Fukushima J. Med. Sci..

[49] Beran J., Lickliter J.D., Schwarz T.F., Johnson C., Chu L., Domachowske J.B., Van Damme P., Withanage K., Fissette L.A., David M.P. (2018). Safety and Immunogenicity of 3 Formulations of an Investigational Respiratory Syncytial Virus Vaccine in Nonpregnant Women: Results From 2 Phase 2 Trials. J. Infect. Dis..

[50] Mankarious S., Lee M., Fischer S., Pyun K.H., Ochs H.D., Oxelius V.A., Wedgwood R.J. (1988). The half-lives of IgG subclasses and specific antibodies in patients with primary immunodeficiency who are receiving intravenously administered immunoglobulin. J. Lab. Clin. Med..

[51] Erlandson S.C., Wang J., Jiang H., Osei-Owusu J., Rockman H.A., Kruse A.C. (2024). Engineering and Characterization of a Long-Half-Life Relaxin Receptor RXFP1 Agonist. Mol. Pharm..

