# Supplementary material for: Anti-Idiotypic Antibody as a Booster Vaccine Against Respiratory Syncytial Virus
Source: Vaccines (Basel). 2025 Jan 2;13(1):35. doi: 10.3390/vaccines13010035 (PMC11768756; doi:10.3390/vaccines13010035)
Supplement: Supplementary file 1 [file vaccines-13-00035-s001.zip › vaccines-3355297-supplementary.pdf]

Supplementary information

MK-1654 Fab preparation and complex formation with Anti-IDs

Protein-protein complex formation:

160 mg of the MK-1654 IgG stock (in-house) was diluted to 6 mg/ml by adding 27 ml of the digestion buffer. 20 ml of papain (Thermo Fisher Scientific, Waltham, MA) was added. The digestion buffer used was prepared fresh and consisted of 10 mM PBS (pH 7.4), 20 mM EDTA, and 17.5 mM Cysteine-HCl (Thermo Fisher Scientific). For the experiment, a total of 100 ml of digestion buffer was prepared. The digestion process was carried out by incubating the mixture in two 50 ml conical tubes for a duration of 5-6 hours. After digestion, protein A purification was performed to isolate the Fab fragments. A 5 ml Hi Trap column (Cytiva, Marlborough, MA) was used, and it was run with PBS (Thermo Fisher Scientific) at a rate of approximately 5 ml/min on an FPLC system. The column was washed with 2 column volumes (2CV) of PBS. The flow-through (FT) and wash fractions were collected to obtain the RB1 Fab fragments. The yield of the Fab fragments was approximately 110 mg (Supplementary Figure S1 a-b).

Complex formation characterization by SEC and Analytical SEC:

RB1 and Anti-ID were mixed at a ratio of 1:1.2 and then subjected to an SEC run using a Hiload 16/60 column (Cytiva, Marlborough, MA). For the bulk loading of Fab-Fab complexes, a sample pump was used. A 4 ml load of 1A6-RB1 was performed at a flow rate of 1 ml/min, while a 3 ml load of 1D4-RB1 was done at a flow rate of 0.5 ml/min. The Anti-ID Fab was added in excess to ensure the identification of the complex peak from the non-complexed excess Fab peak, allowing for the selection of a homogeneous pool of the complex only. An analytical SEC was performed on a Superdex 200 15/150 column where 10 µl of protein at 1 mg/ml was loaded to check for relative elution times, to confirm the complex formation (Supplementary Figure S1c).

1D4 Anti-ID engages with RB1 only via its Light Chains:

All significant interactions between 1D4 Anti-ID and RB1 involve hydrogen bonding, except for one ionic interaction. This is supported by Supplementary Figure 5 and Supplementary Table S2. In the interaction between 1D4 Light Chain (LC) and RB1 Heavy Chain (HC), multiple hydrogen bonding interactions occur. R54 (LFR3) on 1D4 establishes a network of hydrogen bonding interactions with three different residues at the HFR3 of RB1 (Supplementary Figure S5a and Supplementary Table S2). The R71 backbone carbonyl group of RB1 forms a hydrogen bond with the backbone amine group of R54 (Supplementary Figure S5a and Supplementary Table S2), on 1D4. The D73 backbone amine group of RB1 creates a stable hydrogen bond with the carbonyl group of the backbone of R54 (Supplementary Figure S5a and Supplementary Table S2) of 1D4. Moreover, the S74 side chain hydroxyl group of RB1 interacts with the side chain amine group of the guanidium group of R54 on 1D4, forming another hydrogen bond (Supplementary Figure S5a and Supplementary Table S2). The A50 (LCDR2) carbonyl group of 1D4 forms a hydrogen bond with the side chain amine of K57 (HFR3) of RB1 (Supplementary Figure S5b and Supplementary Table S2). Additionally, the Y49 (LFR2) hydroxyl group of 1D4 interacts with the side chain carboxyl group of D73 (HFR3) of RB1 through another hydrogen bond (Supplementary Figure S5b and Supplementary Table S2). Furthermore, the carbonyl group of the side-chain amide of N53 (LFR3) residue of 1D4 interacts with the side-chain amine of K57 (HFR3) of RB1, forming an additional hydrogen bond (Supplementary Figure S5c and Supplementary Table S2). As for the interaction between 1D4 Heavy Chain (HC) and RB1 Heavy Chain (HC), the D101 (HFR4) carboxyl and the carbonyl groups of the side chain on 1D4 interact with K52B (HCDR3) residue of RB1, via an ionic interaction and hydrogen bonding, respectively. This network of hydrogen bonds and the ionic interaction contributes to the overall stability and specificity of the interaction between RB1 and 1D4. Our cryo-EM data explains the reason for the decrease in binding of 1D4 to RB1 upon scrambling of RB1 HCDR3, as there are direct interactions through this RB1 CDR loop. However, we do not see much of the loss of binding of 1D4 to RB1, due to the scrambling of RB1 LCDR3, as there were no physiological interactions found via the light chain of RB1.

However, it remains elusive how, upon scrambling of both the HCDR3 and LCDR3 of RB1, there was a similar impact as that we have seen with the LCDR3 scrambled mutant of RB1 to 1D4. One potential reason could be the non-specific interactions of the RB1 double-scrambled mutant to 1D4, resulting in some non-specific binding. Further investigation may be required to delineate this phenomenon.

RB1 WT, HCDR3 and LCDR3 Scrambled construct sequences with scrambled CDR3 sequences in italics

1) **RB1-WT construct:**

   VH:

   EVQLVESGGGLVRPGRSLRLSCTVSGFSFDDSAMSWVRQAPGKGLEWISFIKSKTYG
   GTKEYAASVKGRFTISRDDSKNIAYLQMNSLKTEDTAVYYCTRGAPYGGNSDYYYGLD
   VWGQGTTVTVSS

   VK:

   DIQMTQSPSSLSASVGDRVTITCRTSQDVRGALAWYQQKPGKAPKLLIFDASSLETGVP
   SRFSGSGSGTVFTLTISSLQPEDFAAYYCQQFLDFPFTFGQGTRLEIK

2) **RB1-HCDR3 scrambled construct:**

   VH:

   EVQLVESGGGLVRPGRSLRLSCTVSGFSFDDSAMSWVRQAPGKGLEWISFIKSKTYG
   GTKEYAASVKGRFTISRDDSKNIAYLQMNSLKTEDTAVYYCTRGVADPLYGGYGYNYD
   SWGQGTTVTVSS

   VK:

   DIQMTQSPSSLSASVGDRVTITCRTSQDVRGALAWYQQKPGKAPKLLIFDASSLETGVP
   SRFSGSGSGTVFTLTISSLQPEDFAAYYCQQFLDFPFTFGQGTRLEIK

3) **RB1-LCDR3 scrambled construct:**

VH:

EVQLVESGGGLVRPGRSLRLSCTVSGFSFDDSAMSWVRQAPGKGLEWISFIKSKTYG

GTKEYAASVKGRFTISRDDSKNIAYLQMNSLKTEDTAVYYCTRGAPYGGNSDYYYGLD

VWGQGTTVTVSS

VK:

DIQMTQSPSSLSASVGDRVTITCRTSQDVRGALAWYQQKPGKAPKLLIFDASSLETGVP

SRFSGSGSGTVFTLTISSLQPEDFAAYYCQTQFFPLFDFGQGTRLEIK

4) **RB1- HCDR3-LCDR3 scrambled construct:**

VH:

EVQLVESGGGLVRPGRSLRLSCTVSGFSFDDSAMSWVRQAPGKGLEWISFIKSKTYG

GTKEYAASVKGRFTISRDDSKNIAYLQMNSLKTEDTAVYYCTRGVADPLYGGYGYNYD

SWGQGTTVTVSS

VK:

DIQMTQSPSSLSASVGDRVTITCRTSQDVRGALAWYQQKPGKAPKLLIFDASSLETGVP

SRFSGSGSGTVFTLTISSLQPEDFAAYYCQTQFFPLFDFGQGTRLEIK

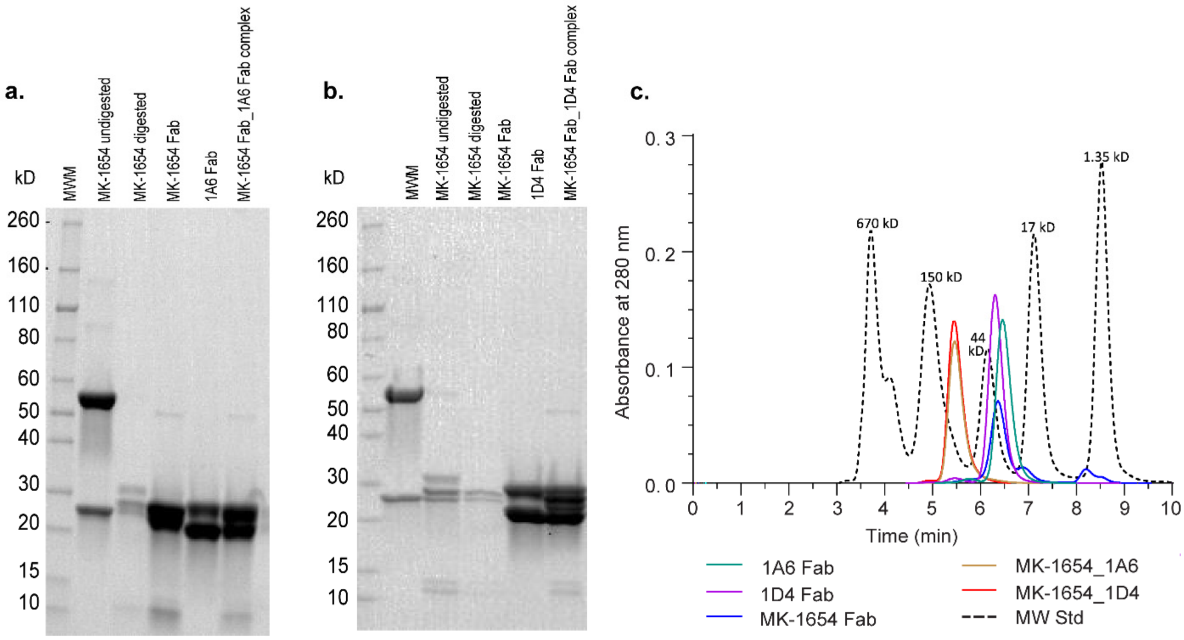

Supplementary Figure S1. Protein sample quality assessment for Cryo-EM. (a-b.) Representative SDS protein gel showing the sample purity for the MK-1654 Fab-1A6 Fab Complex and the MK-1654 Fab-1D4 Fab Complex, respectively. **c.** Representative analytical gel filtration profile demonstrating the sample preparation for Cryo-EM analysis.

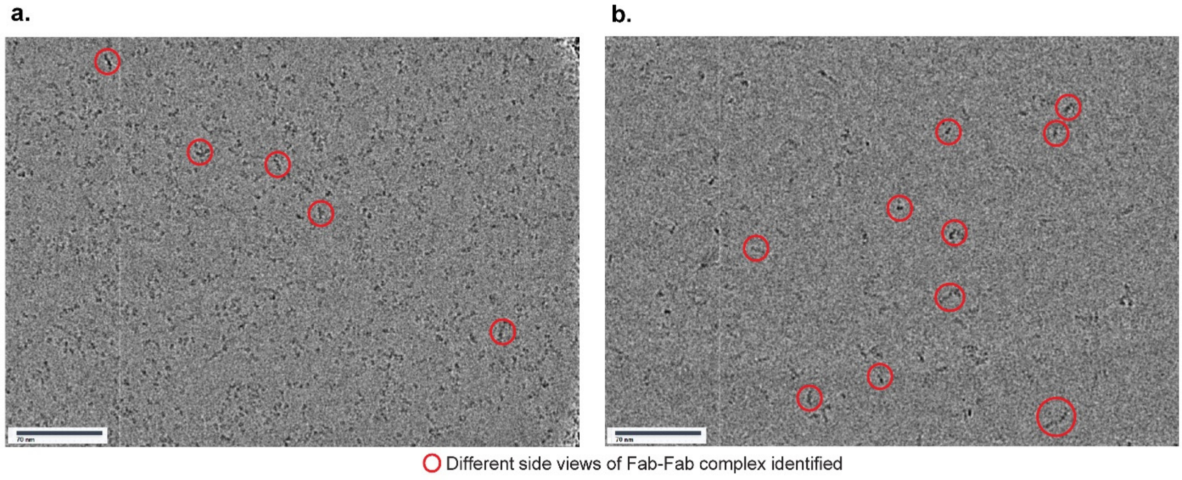

Supplementary Figure S2. Fab-Fab particles Observed In the Field of View. a) 1A6-RB1 complex electron micrograph. **b)** 1D4-RB1 complex electron micrograph. Some example particles from different side views of the Fab-Fab complex identified are marked in red circles.

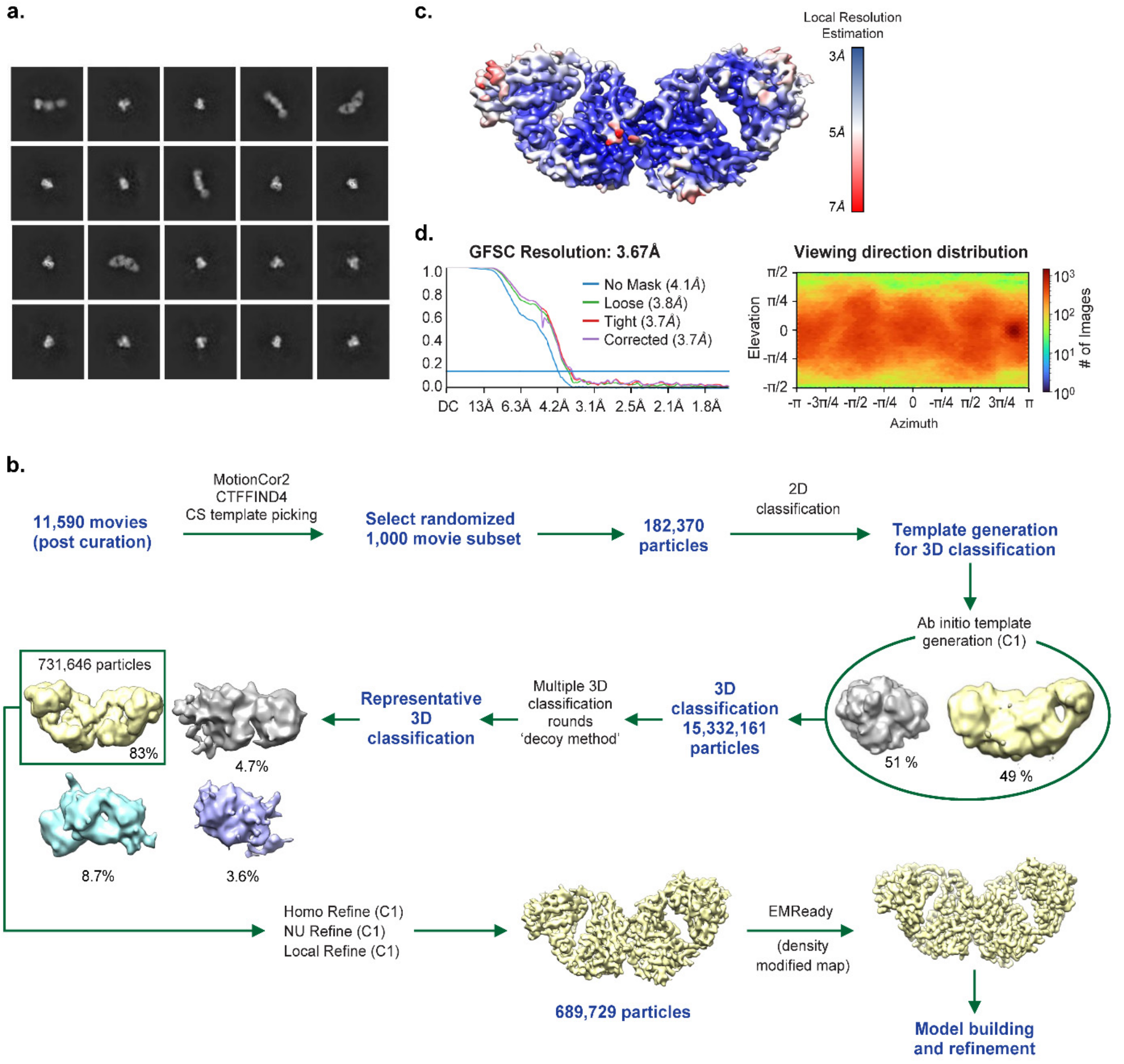

Supplementary Figure S3. 1A6-RB1 complex cryo-EM data processing. a) Example 2D class averages for 1A6-RB1 complex. **b)** Single-particle cryo-EM, data processing scheme, using cryoSPARC. **c)** Density maps colored by local resolution for 1A6-RB1 complex. **d)** Gold standard Fourier-Shell correlation resolution plots with a cut-off at 0.143 indicated by a blue line angular distribution of particles included in the final cryo-EM reconstruction.

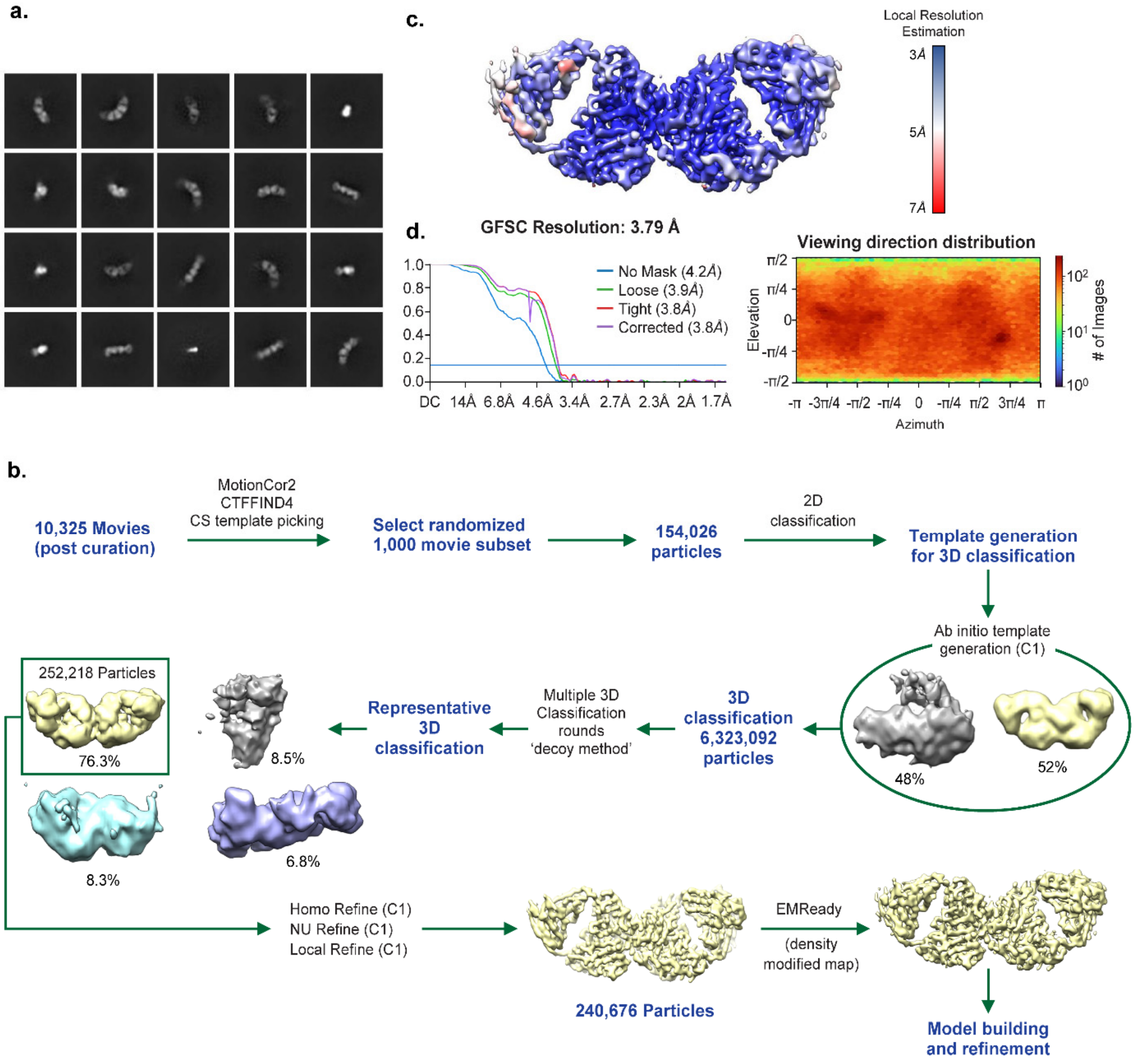

Supplementary Figure S4. 1D4-RB1 complex cryo-EM data processing. a) Example 2D class averages for 1D4-RB1 complex. **b)** Single-particle cryo-EM, data processing scheme, using cryoSPARC. **c)** Density maps colored by local resolution for 1A6-RB1 complex. **d)** Gold standard Fourier-Shell correlation resolution plots with a cut-off at 0.143 indicated by a blue line angular distribution of particles included in the final cryo-EM reconstruction.

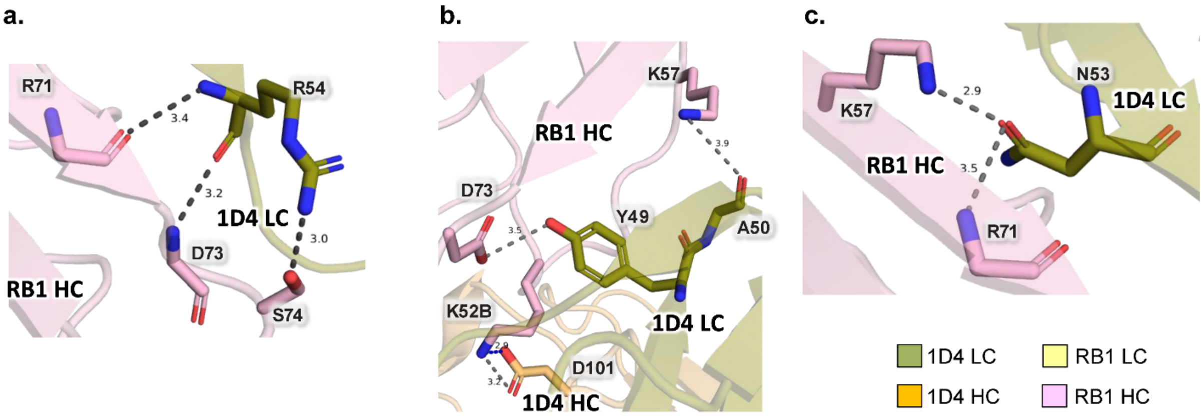

Supplementary Figure S5: 1D4 Anti-ID Light chain (LC) and Heavy chain (HC) interact only with RB1 HC. a-c.) RB1 HC (light pink) interacts with 1D4 LC (deep olive) via hydrogen bonding (gray dashed lines) with the estimated bond distances (in Å) measured using PyMOL, B. 1D4 HC (sand) with RB1 HC (light pink) via one salt bridge formation (blue dashed lines), with the corresponding estimated bond distance (in Å).

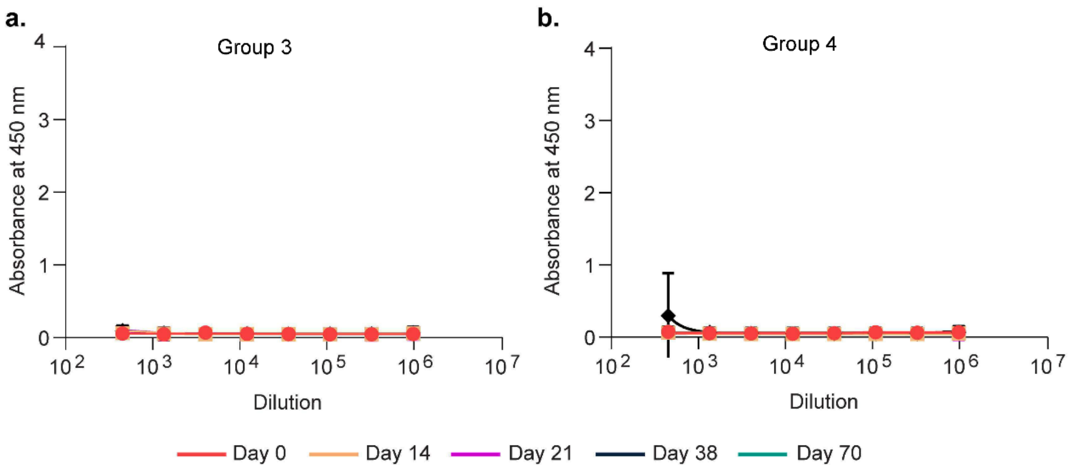

Supplementary Figure S6. Anti-RSV F titer boost for 1A6 x2-RSV F groups that were primed with different doses of 1A6 Fab. a,b, 10 µg and 50 µg of 1A6 Anti-ID Fab, respectively, for Group 3 and 4 animals.

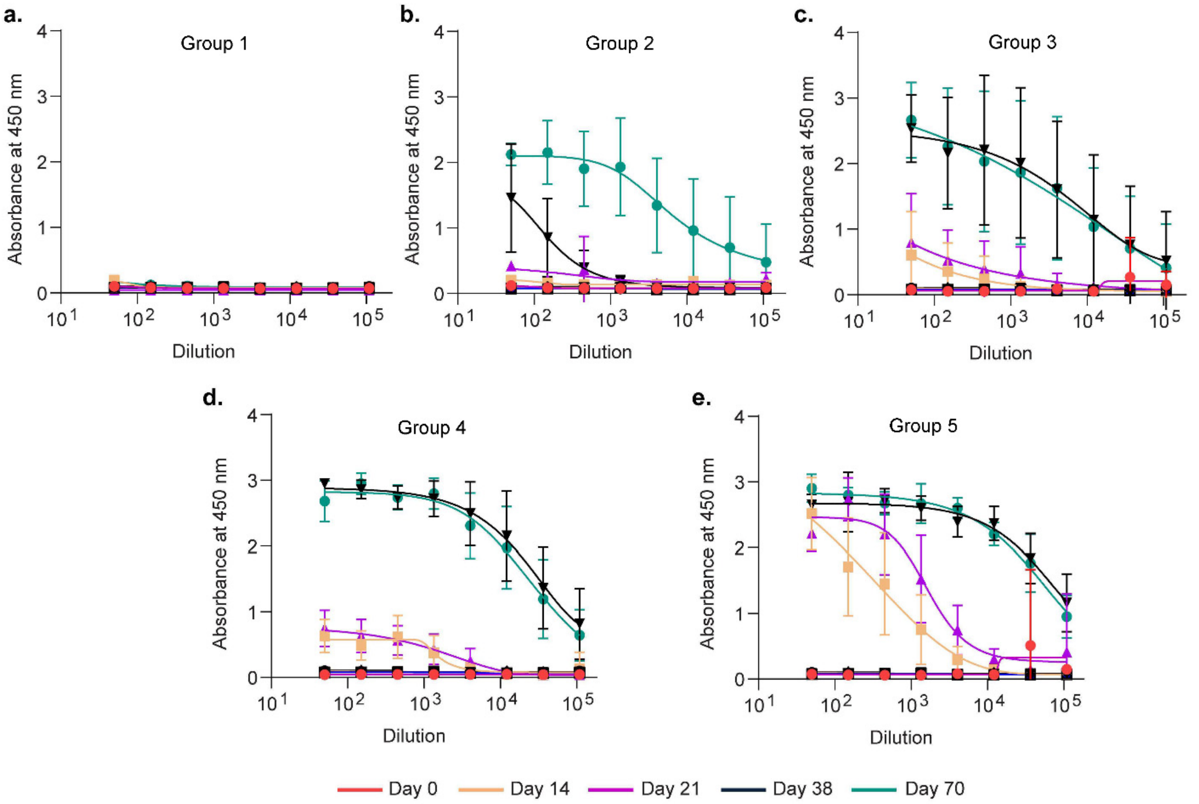

Supplementary Figure S7. Mean Anti-Fab titer boost for animals from Groups 1-5 on different bleeds. a,b) RSV Fx3, RSV F 1A6 x2, **c-e)** 1A6 x2 RSV F at 10 µg, 50 µg, and 100 µg 1A6 Fab as prime and booster 1, respectively.

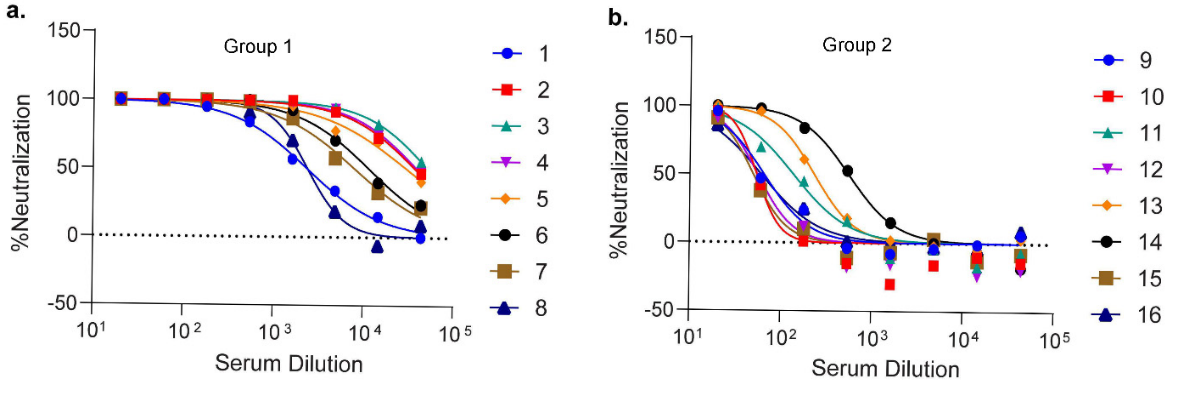

Supplementary Figure S8. Anti-IDs Boost Anti-RSV Neutralization Titer. a-b. Anti-RSV titer boosts for individual sera from animals of groups RSV Fx3 and RSV F 1A6 x2, respectively, from the terminal bleeds. The dotted line indicates the limit of detection for the experiment.

Supplementary Table S1: Cryo-EM data collection, refinement, validation, and statistics

	**1A6 Fab - RB1 Fab (EMD- 48393) (PDB 9MML)**	**1D4 Fab - RB1 Fab (EMD- 48404 (PDB 9MMV)**
**Data collection and processing**		
Microscope	Titan Krios	Titan Krios
Voltage (kV)	300	300
Magnification	105,000	105,000
Pixel size (Å)	0.815	0.815
Electron Exposure (e^-/A^2)	50	50
Defocus Range (μm)	-0.6 to -2.2	-0.6 to -2.3
Detector	Gatan K3	Gatan K3
Movies	11,590	10,325
Symmetry imposed	C1	C1
No. of particles after 2D classification	N/A	N/A
No. of particles in final structure	689,729	240,676
Map resolution (Å) at 0.143 FSC criterion	3.67	3.79
Sharpening *B* factor (A^2)	-138.7	-123.7
Processing Software	CryoSPARC	CryoSPARC
**Refinement**		
Initial Model Used	6OUS	6OUS
Model resolution (Å)	3.67	3.79
FSC threshold	0.143	0.143
Model composition		
No. of Atoms Macromolecules	3506	3439
Ligands	NA	NA
Model to map fit	0.8821	0.8762
B factors ($Å^2$)		
Average B-factor Macromolecule	59.6	63.7
Ligand	NA	NA
Bond Lengths (Å)	0.007	0.007
Bond angles (°)	0.851	0.97
Validation		
MolProbity score	2.30	2.03
Clashscore	17.29	13.22
Poor rotamers (%)	1.85	1.08
Ramachandran Plot		
Favored (%)	94.74	94.62
Allowed (%)	5.26	5.16
Disallowed (%)	0	0.22

Supplementary Table S2: 1D4 interaction with RB1

1D4 Site	Residue	RB1 Site	Residue	ΔG (kcal/mole)
LCDR2	A50	HFR3	K57	-1.1
LFR2	Y49		D73	-1.4
LFR3	N53		R71	-1.5
	R54		R71	-2.1
	R54		D73	-4.7
	R54		S74	-2.1
	N53		K57	-9.3
HFR4	D101		K52B	-31.71

ΔG calculations were done on MOE; LCDR = VL CDR region, HCDR = VH CDR region, LFR = VL Framework region, HFR = VH Framework region

Supplementary Table S3: Individual animal terminal bleed (Day70) sera IC50

RSV F x3 – Day 70 Individual animal sera								
IC50 (dilution)	1	2	3	4	5	6	7	8
	2389	>43740	>43740	>43740	>43740	11643	8091	2405
RSV F-1A6 x2 – Day 70 Individual animal sera								
IC50 (dilution)	9	10	11	12	13	14	15	16
	65.57	54.45	138.1	55.72	234.3	559.3	49.92	60.81
